# An Introduction to the *Toxins* Special Issue on “Novel Pharmacological Inhibitors for Bacterial Protein Toxins”

**DOI:** 10.3390/toxins9050160

**Published:** 2017-05-11

**Authors:** Holger Barth

**Affiliations:** Institute of Pharmacology and Toxicology, University of Ulm Medical Center, Albert-Einstein-Allee 11, 89081 Ulm, Germany; holger.barth@uni-ulm.de

Bacterial AB-type protein toxins that consist of an enzymatically active subunit (A) and a binding/transport subunit (B), are among the most toxic substances and represent the causative agents for a variety of severe human and animal diseases, such as in the context of infections, post-traumatic complications or food poisoning. Moreover, some AB-type toxins can be misused as biological warfare agents and in the context of bio-terrorism activities. Therefore, novel pharmacological inhibitors against such toxins are urgently needed. The remarkable toxicity of AB-type toxins is due to their unique modular structure and mode of action. Their B subunit very efficiently mediates the transport of the A subunit into the cytosol of mammalian target cells where the A subunit modifies its specific substrate molecules resulting in cell damage. Thus, such toxins are highly potent as well as being very substrate-specific enzymes that act inside cells, thereby causing the characteristic diseases associated with the individual toxins and toxin-producing bacteria. In past years, significant progress has been made in understanding the molecular mechanisms underlying the cellular uptake of bacterial AB-type toxins. This process involves receptor-binding, receptor-mediated endocytosis, intracellular transport in vesicles and finally the translocation of the enzyme subunit across cellular membranes into the cytosol. It was discovered that some toxins form pores in endosomal membranes and exploit host cell factors, such as chaperones, for the transport of their A subunit across intracellular membranes into the cytosol. This translocation can occur either from acidified endosomes, as first reported for Diphtheria toxin, or later in the cell from the endoplasmic reticulum, as described originally for Cholera toxin and other AB_5_ toxins, including the Pertussis toxin and Shiga toxin.

Increasing knowledge about the cellular uptake has allowed for the development or screening of pharmacological compounds that inhibit the individual steps of toxin uptake and protect cells from intoxication, which is described in various chapters of this book. For all these inhibitors, the final consequence is the same and independent of which state of toxin uptake or transport in the cell they prevent. As long as the A subunit does not reach the cytosol, the cytotoxic effects do not occur if the substrate for the respective toxin remains in the cytosol. In conclusion, compounds that act on the level of the toxins rather than on the toxin-producing bacteria should have attractive therapeutic perspectives, including the following:

▪ They can serve as anti-toxins when only the toxins, but not the toxin-producing bacteria, are taken up and enter the body, such as botulinum neurotoxin (food poisoning).
▪ During infections, they would inhibit the mode of action of the toxins, which are already released by the bacteria and therefore could be combined with antibiotics. This should be of particular interest when the infection is caused by toxin-producing bacteria which are (multi-)resistant towards the classical antibiotics.
▪ In contrast to the toxin-neutralizing antibodies (antisera), they act on toxins that are already internalized into their target cells, as long as the A subunit did not reach the cytosol. Neutralizing antibodies are still not available for each toxin.
▪ Some compounds inhibit cellular uptake of toxin families (e.g., Adenosine diphosphate (ADP)-ribosyltransferases, pore-forming toxins) and therefore could serve as potential therapeutics against all toxins that are members of such toxin families.

As it becomes evident from the various chapters of this book, many different types of inhibitors have been identified and their mode of action has been analyzed in detail on the biochemical and cellular levels. Most of the presented research includes in vitro data with cell-based test systems, animals or human organoids. Thus, further efforts are required to transfer this promising novel knowledge into therapeutic strategies.

The first chapter of this book from Kirsten Sandvig’s group provides an excellent overview on the uptake and intracellular transport of bacterial toxins with the focus on the Shiga toxins. These AB_5_ toxins are produced by various bacteria and associated with severe human diseases, such as the hemolytic-uremic syndrome (HUS). In this chapter, a variety of pharmacological inhibitors are reviewed, which inhibit each individual step during the cell entry of Shiga toxins from receptor-binding to translocation of the A subunit from the endoplasmic reticulum into the host cell cytosol [1].

In the second chapter, the Cheng group shows that treatment with several probiotic micro-organisms, including Saccharomyces and Lactobacillus strains, but not with a non-probiotic strain of *Escherichia coli*, inhibits the internalization of *Clostridium botulinum* neurotoxin serotype A (BoNT/A) into human epithelial cells (CaCo-2) in vitro. The probiotic strains do not bind BoNT A or cause its degradation, although the findings suggest that there is some competition between the strains and BoNT/A for binding to the cell surface [2]. 

The third chapter from Bruce McClane’s group (Li et al.) suggests sialidase inhibitors as potential novel therapeutics to treat/prevent intestinal infections caused by *Clostridium perfringens*. *C. perfringens* causes histotoxic infections with traumatic gas gangrene and myonecrosis as well as intestinal infections of humans and animals. *C. perfringens* produces up to three sialidases that are involved in the histotoxic effects. The sialidases may also contribute to the intestinal infection, because they upregulate the production of some toxins, their activity and their binding to the surface of target cells. Moreover, there is some evidence that the sialidase, NanI, might contribute to the intestinal colonization by *C. perfringens*, because strains that cause acute food poisoning lack the NanI gene while strains that cause chronic intestinal infections carry this gene [3]. 

The fourth chapter from the Genth group [4] and fifth chapter from the Barth laboratory [5] both describe compounds that inhibit the cellular uptake of single chain AB-type toxins, namely the *Clostridium sordellii* Lethal toxin and Diphtheria toxin, respectively. After their receptor-mediated endocytosis, both toxins deliver their A subunit from acidic endosomes into the cytosol. Schelle and co-workers identified the p38_alpha/beta_ MAP kinase inhibitor SB203580 as an inhibitor against the Lethal toxin and found that this compound might inhibit the toxin uptake rather than the enzymatic reaction in the cell, because it inhibits the Lethal toxin but not the related TcdB from *C. difficile*, which has the same enzymatic mode of action. Schnell et al. identified EGA, a semicarbazone compound, as a potent inhibitor against Diphtheria toxin and showed that EGA prevents the pH-dependent transport of the A subunit of this toxin across cell membranes. 

The last two chapters focus on compounds that block the heptameric trans-membrane pores formed by the B subunits of binary toxins from *Bacillus anthracis* in addition to the C2 and iota toxins from *Clostridium botulinum* and *C. perfringens*, respectively, in endosomal membranes under acidic conditions. These pores serve as translocation channels for the A subunits of these toxins, which unfold to translocate through the pores across endosomal membranes into the host cell cytosol. Pharmacological pore-blockers prevent this step and protect cells from intoxication by anthrax and clostridial binary toxins. The chapter from the Nestorovich group [6] describes pore-blockers, which are directed against the PA63 pore of the anthrax toxins and are based on cyclic dendrimers. These compounds efficiently block the pores formed by the PA63 component of anthrax toxin in artificial lipid bilayer membranes, which enables single molecule analysis in biophysical in vitro approaches. The Benz and Barth groups [7] characterized compounds derived from chloroquine, which inhibit the heptameric trans-membrane pores of the binary clostridial C2 and iota toxins in lipid bilayers in vitro, and demonstrated the protective effects of the most efficient pore-blockers in cell-based experiments. 
